# Experimental hut evaluation of bednets treated with an organophosphate (chlorpyrifos-methyl) or a pyrethroid (lambdacyhalothrin) alone and in combination against insecticide-resistant *Anopheles gambiae *and *Culex quinquefasciatus *mosquitoes

**DOI:** 10.1186/1475-2875-4-25

**Published:** 2005-05-26

**Authors:** Alex N Asidi, Raphael N' Guessan, Alphonsine A Koffi, Christopher F Curtis, Jean-Marc Hougard, Fabrice Chandre, Vincent Corbel, Frédéric Darriet, Morteza Zaim, Mark W Rowland

**Affiliations:** 1 London School of Hygiene & Tropical Medicine, London, WC1E 7HT, UK; 2 Centre Pierre Richet, 01 PO Box 1500, Bouaké 01, Côte d'Ivoire, France; 3 Laboratoire de Lutte contre les Insectes Nuisibles (LIN), 911 Avenue Agropolis, PO Box 64501, 34394 Montpellier Cedex 5, France; 4 World Health Organization, 27 Avenue Appia, CH-1211 Geneva 27, Switzerland

## Abstract

**Background:**

Pyrethroid resistant mosquitoes are becoming increasingly common in parts of Africa. It is important to identify alternative insecticides which, if necessary, could be used to replace or supplement the pyrethroids for use on treated nets. Certain compounds of an earlier generation of insecticides, the organophosphates may have potential as net treatments.

**Methods:**

Comparative studies of chlorpyrifos-methyl (CM), an organophosphate with low mammalian toxicity, and lambdacyhalothrin (L), a pyrethroid, were conducted in experimental huts in Côte d'Ivoire, West Africa. *Anopheles gambiae *and *Culex quinquefasciatus *mosquitoes from the area are resistant to pyrethroids and organophosphates (*kdr *and insensitive acetylcholinesterase *Ace.1*^*R*^). Several treatments and application rates on intact or holed nets were evaluated, including single treatments, mixtures, and differential wall/ceiling treatments.

**Results and Conclusion:**

All of the treatments were effective in reducing blood feeding from sleepers under the nets and in killing both species of mosquito, despite the presence of the *kdr *and *Ace.1*^*R *^genes at high frequency. In most cases, the effects of the various treatments did not differ significantly. Five washes of the nets in soap solution did not reduce the impact of the insecticides on *A. gambiae *mortality, but did lead to an increase in blood feeding. The three combinations performed no differently from the single insecticide treatments, but the low dose mixture performed encouragingly well indicating that such combinations might be used for controlling insecticide resistant mosquitoes. Mortality of mosquitoes that carried both *Ace.1*^*R *^and *Ace.1*^*S *^genes did not differ significantly from mosquitoes that carried only *Ace.1*^*S *^genes on any of the treated nets, indicating that the *Ace.1*^*R *^allele does not confer effective resistance to chlorpyrifos-methyl under the realistic conditions of an experimental hut.

## Background

Insecticide-treated nets (ITN) are an important component of the Roll Back Malaria campaign to reduce malaria morbidity and mortality in Africa. Pyrethroids are the only group of insecticides currently recommended for use on nets [1]. In recent years, pyrethroid resistance has become widespread among anopheline mosquitoes in western Africa and has also arisen in eastern and southern Africa [2-6]. Pyrethroid resistance has evolved concurrently in the filariasis vector and nuisance mosquito *Culex quinquefasciatus *[7,8].

Initial alarm over the rapid spread of the *kdr *allele responsible for pyrethoid resistance [9] has been tempered by recent evidence which indicates that nets incorporating permethrin continue to reduce malaria transmission and morbidity in an area known to have a high frequency of *kdr *[10]. Experimental hut studies in a similar area confirm that pyrethroid treated nets continue to kill pyrethroid resistant mosquitoes [11-14]. However, in another PCR genotyping hut study by Kolaczinski et al [15] showed a significantly higher frequency of the *kdr *gene among survivors of alphacypermethrin- or etofenprox-treated nets than in mosquitoes which were killed by these treatments. In Kenya, a different form of the *kdr *gene was found in *Anopheles gambiae *by Ranson et al [5], in an area where one of the most successful large-scale ITN trials was subsequently carried out [16,17]. These findings may allay initial fears but it would be complacent to assume that pyrethroid-treated nets will remain effective indefinitely. Selection of supplementary resistance mechanisms could tip the balance towards control failure [3,18,19], as happened with indoor residual spraying in South Africa owing to selection of a metabolic form of resistance in *Anopheles funestus *which required switching back to DDT to restore malaria control [6]. The danger of pyrethroid resistance is apparent in Tanzanian *C. quinquefasciatus*. It effectively prevents mortality with pyrethroid treated nets in experimental huts [20] and prevents *Culex *population suppression when ITNs are used by whole communities, in contrast to high mortality and population suppression of susceptible *A. gambiae *populations under these conditions [21].

Finding an alternative to the pyrethroids has, therefore, become a priority [22]. Some members of the earlier generation of insecticides, the organophosphates (OPs) and carbamates, although developed primarily for agricultural use and for indoor residual spraying, may have potential as net treatments. If there is a complete lack of cross-resistance to pyrethroid resistant mosquitoes, use of such compounds in combination with pyrethroids may provide an opportunity for resistance management [23,24].

Experimental hut studies have shown that the performance of the carbamate carbosulfan on nets against pyrethroid- resistant *Anopheles *and carbamate- resistant *Culex *mosquitoes is equivalent or better than that of pyrethroids against susceptible mosquitoes [14,15]. Because of a perceived risk of human toxicity with a carbosulfan breakdown product, carbofuran, some doubt has been cast over the suitability of carbosulfan as a net treatment [13,25]. Chlorpyrifos-methyl (Reldan^®^, Dow AgroSciences), an OP used in agriculture to control stored product pests, may be more suitable, being classified by WHO as unlikely to present acute hazard in normal use, whereas carbosulfan and the majority of pyrethroids recommended for treatment of mosquito nets are classified by WHO as Class II, i.e. moderately hazardous [26].

Broad spectrum resistance to organophosphates and carbamates caused by insensitive acetylcholinesterase mechanisms have been identified in *A. gambiae *and *C. quinquefasciatus *from Côte d'Ivoire [4,27,28]. To assess the potential of chlorpyrifos-methyl to control resistant anophelines and culicines, the performance of nets treated with chlorpyrifos-methyl, lambdacyhalothrin and various combinations of the two was compared in experimental huts near Bouaké, Côte d'Ivoire.

## Materials and methods

### Study area and experimental huts

The treated nets were evaluated in 11 experimental huts at a field site of the Institut Pierre Richet in Yaokoffikro, Bouaké, Côte d'Ivoire. The huts were situated near rice and vegetable fields and arrayed in two rows with a 5 metre gap between huts. The style of the hut was typical of the region. Each was made from concrete bricks, with a corrugated iron roof, a ceiling of thick polyethylene sheeting, and a concrete base surrounded by a water-filled channel to prevent entry of ants [11]. Mosquito access was via 4 window slits constructed from pieces of plywood, fixed at an angle to create a funnel with a 1 cm wide gap. Mosquitoes had to fly upward to enter through the gap and downwards to exit; this precluded or, at worst, limited exodus though the aperture enabling the majority of entering mosquitoes to be accounted for. A verandah trap made of polyethylene sheeting and screening mesh measuring 2 m long, 1.5 m wide, and 1.5 m high, projected from the back wall of each hut. Movement of mosquitoes between hut and verandah was unimpeded. All huts were thoroughly cleaned before the trial. Sheets were laid over the floor each night to ease the collecting of knocked down mosquitoes in the morning.

### Bednets

The nets were made from white 100-denier polyester (SiamDutch Mosquito Netting Co., Thailand). They measured 1.9 m long, 1.8 m wide and 1.5 m high, and had a surface area of 14.5 m^2^. To simulate badly torn nets, 80 holes, each measuring 2 × 2 cm, were cut in the sides and ends of all but two of the nets.

### Insecticide treatment

The insecticides used were:

chlorpyrifos-methyl 38.8% CS ('Reldan GF934', Dow AgroSciences), an experimental microencapsulated suspension, designed for controlled residual activity and short outdoor persistence).

lambdacyhalothrin 2.5% CS, ('Icon', Syngenta UK), a commercial microencapsulated formulation.

Impregnation of nets was carried out using the formula of Pleass et al [29] to calculate the amount of insecticide needed. The nine treatments and target application rates were:

lambdacyalothrin 18 mg/m^2^, holed, unwashed

lambdacyalothrin 18 mg/m^2^, holed, washed with soap 5 times

chlorpyrifos-methyl 100 mg/m^2^, holed, unwashed

chlorpyrifos-methyl 100 mg/m^2^, intact, washed with soap 5 times

chlorpyrifos-methyl 250 mg/m^2^, holed, unwashed

chlorpyrifos-methyl 250 mg/m^2^, holed, washed with soap 5 times

mixture of chlorpyrifos-methyl 100 mg/m^2 ^and lambdacyalothrin 18 mg/m^2^, holed, unwashed

mixture of chlorpyrifos-methyl 25 mg/m^2 ^and lambdacyalothrin 4.5 mg/m^2^, holed, unwashed

chlorpyrifos-methyl 100 mg/m^2 ^on ceiling of net and lambdacyalothrin 18 mg/m^2 ^on walls, holed, unwashed. Differential treatment of the ceiling and walls of a net has been called "two-in-one" by Guillet et al [25] and a "mosaic" by others [13]. However, the term mosaic is conventionally used to describe a resistance management strategy in which two or more chemicals are used for spraying different sectors of countryside, the sectors being large enough to contain their "own" mosquito populations in which different resistance gene frequencies would evolve as a result of different selection pressures, with only limited exchange of genes by immigration. Thus, in this paper we have preferred to adopt the term "two-in-one", suggested by Guillet et al [25].

untreated control, intact

untreated control, holed.

The application rate of 18 mg/m^2 ^lambdacyalothrin was the same as that used by Asidi et al [14] and was in the application dose range proposed by WHO. The 100 mg/m^2 ^chlorpyrifos-methyl (CM) treatment was twice the dosage needed to kill 100% of a laboratory OP resistant strain (DUBAI 234) in a 3 min exposure bioassay test. To assess the effect of repeated washing, a higher application rate of CM was used initially (250 mg/m^2^). Washing was done by hand using a palm-oil soap 'Maxi Mousse', and was repeated five times with a one-day interval between washes. The effect of holed versus intact nets was assessed using untreated and 100 mg/m^2 ^CM treatments. The mixture of lambdacyhalothin and chlorpyrifos-methyl was prepared with the two CS formulations mixed in water. The intention with the low dose mixture (25 mg/m^2 ^CM and 4.5 mg/m^2 ^lambdacyhalothin) was to examine whether efficacy would be maintained after a period of simulated insecticide decay or whether differential decay might lead to selection of *Ace.1*^*R *^or *kdr *resistance genotypes. The "two-in-one" net was prepared by cutting the ceiling from the walls of the net, dipping the two sections in chlorpyrifos-methyl and lambdacyhalothin respectively, and then sewing the two sections together again. Untreated intact and holed nets were used as controls.

### Sleepers and mosquito collections

The treatments were randomly allocated to the eleven experimental huts. Eleven adult men were paid to sleep in the huts each night from 20.00 to 05.00 hours and to collect mosquitoes in the mornings. The sleepers/collectors were experienced in collecting mosquitoes, gave informed consent and were given malaria chemoprophylaxis.

The trial ran for only 33 nights over 6 weeks (from 15 August 2002). The trial was planned to run for 44 nights but had to be curtailed owing to political unrest. The sleepers were rotated between huts to correct for possible variation in individual attractiveness. The nets were not rotated for fear of cross-contaminating huts with different treatments. There was a risk in this experimental design of not being able to separate possible confounding factors due to variation in hut attractiveness independent from that of treatment. However, baseline measurements indicated that the huts were comparable in attractiveness (Table 1). Sleepers were questioned each day during the first two weeks to find out whether they experienced any side effects from using nets.

**Table 1:** Mean numbers of mosquitoes collected per night over 10 nights before installation of treated nets.

Hut number	*An. gambiae*	Other anophelines	*Culex *spp.	*Aedes *spp.	*Mansonia *spp.	Overall means
1	4.2	0.2	3.9	0.6	5.2	14.1
2	5.3	0.1	2.7	0.8	3.9	12.9
3	6.5	0.6	1.6	0.5	5.4	14.6
4	5.1	0.3	2.9	0.6	3.8	12.7
5	6.1	0.4	2.8	1.0	3.6	13.9
6	5.4	0.5	1.9	0.3	3.9	12.0
7	5.1	0.5	2.6	0.2	4.4	12.8
8	4.9	0.6	0.9	0.6	4.8	11.8
9	6.2	0.4	2.6	0.6	3.7	13.5
10	6.5	0.2	3.8	0.5	3.3	14.4

Each dawn, the huts were searched and all mosquitoes were collected from the floors, walls, and ceilings of rooms, verandahs and nets. Mosquitoes were identified and scored as blood-fed or unfed and dead or alive. Male mosquitoes were not recorded. Live females were held in netted plastic cups and supplied with 10% honey solution for 24 h before recording any delayed mortality. All *A. gambiae *and *C. quinquefasciatus *were kept for determination of *kdr *and *Ace.1*^*R *^genotypes.

### DNA diagnostic test for the pyrethroid resistance kdr and insensitive acetylcholinesterase G119S mutations in single A. gambiae

Genomic DNA was extracted from single mosquitoes according to Collins et al [30]. For determination of acetylcholinesterase mutation, the DNA was PCR amplified with the degenerate primers Moustdir1 5'CCGGGNGCSACYAT-GTGGAA3' and Moustrev1 5'ACGATMACGTTCTCYTCCGA3' for thirty cycles (94°C for 30 seconds, 52°C for 30 seconds and 72°C for 1 minute), the PCR fragments were then digested with *Al*uI restriction enzyme according to the manufacturer's instructions and fractionated on a 2% agarose gel according to Weil et al [31]. Genotypes for pyrethroid resistance *kdr *"leu-phe" mutation were determined according to Martinez-Torres et al [9].

### Data analysis

The effect of each treatment was assessed relative to the control in terms of deterrency (the number of mosquitoes caught in each hut), excito-repellency (the proportion of mosquitoes in the verandah traps), blood feeding inhibition and mortality rates. Proportional data were analysed using logistic regression (STATA 6 software). Comparisons between treatments were made by successively dropping treatments from the overall comparison and this process allowed each treatment to be compared with every other. Owing to non-normality of the data the numbers of blood-fed and dead mosquitoes and overall totals collected from each hut were compared using Wilcoxon rank sum non-parametric tests. Genotype frequencies were tested using χ^2 ^or Fisher's exact test.

## Results

### Mosquito abundance

To assess any difference in the attractiveness of the huts to mosquitoes, preliminary collections were carried out over 100 hut-nights (10 huts × 10 nights) from late July 2002, with the sleepers/collectors being rotated between huts on successive nights (the 11th hut was being used for another purpose at this time). A total of 1328 mosquitoes were recorded of which 41.6% were *A. gambiae*, 19.4% *Culex *spp., 31.6% *Mansonia *spp., 4.3% *Aedes *spp., 2% *A. funestus*, 0.9% *Anopheles pharaonsis*, 0.5% *Anopheles coustani*, 0.2% *Coquillettidia crystata *and 0.1% *Eretmapodites*. There were no significant differences between the huts in the numbers of each species collected (F = 0.411 df = 9,9 *P *= 0.926) (Table 1).

### Efficacy of treatment

From 363 hut-nights collections (33 days × 11 huts) a total of 5,639 mosquitoes were recorded of which 10% were *A. gambiae *and 45% *Culex *spp. (*C. quinquefasciatus *predominated). The markedly lower *A. gambiae *frequency than that mentioned in the previous paragraph was presumably due to a seasonal change. Of the remaining species 23% were *A. coustani*, 35% *Mansonia *spp., 6% *Aedes *spp., 0.4% *A. funestus*, and 1% *A. pharaoensis *with *Coquillettidia crystata *and *Eretmapodites *being present in small numbers. Only the malaria vector *A. gambiae *and the nuisance mosquito *Culex *spp. were subjected to analysis. Additional file 1 and Additional file 2 summarize the data for *A. gambiae *and *Culex *spp., respectively. All types of insecticide treatment appeared to deter entry of mosquitoes into huts, compared to that of untreated nets. Estimated deterrency among *A. gambiae *ranged from 30% with the low dosage mixture to 71% with lambdacyhothrin. However, the differences between treatments never reached statistical significance among the relatively small numbers of *A. gambiae *caught. Percentage deterrency among *Culex *spp. was less than the estimates for *A. gambiae*, but was more often statistically significant among the larger numbers caught. Estimated deterrency of *Culex *spp. ranged from 36% with the washed chlorpyrifos-methyl treated net to 58% with the unwashed lambdacyhalothrin net but the differences between unwashed and washed nets were not significant.

Additional file 1 and Additional file 2 show data on mosquito feeding and mortality expressed either as mean numbers per daily collection (cols. 9 and 12) or as percentages among those that entered the huts (cols. 6–8 and 10–11).

Untreated nets failed to give protection when holed: 39% of *A. gambiae *and 25% of *Culex *spp. were blood-fed, whereas with the intact net only 7% of *A*. *gambiae *and 2% of *Culex *were blood-fed. Treatment with chlorpyrifos-methyl or lambdacyhalothrin restored the capacity of holed nets to inhibit blood feeding. After five washes of the lambdacyhalothrin (18 mg/m^2^) or chlorpyrifos-methyl (250 mg/m^2^) treated nets, the inhibition of blood feeding shown by in *A. gambiae *was no longer statistically significant. Among the *Culex*, on the other hand, blood feeding remained significantly inhibited after washing, both for lambdacyhalothrin and chlorpyrifos-methyl treated nets. The proportion of *A. gambiae *and *Culex *killed by lambdacyhalothrin or chlorpyrifos-methyl treatments were similar before and after washing, indicating that the treatments remained highly insecticidal even after 5 washes.

The treatments deterred *A. gambiae *from entering the holed nets. The proportions collected from inside the nets were 20.5% for the untreated nets, 10.0% for the chlorpyrifos-methyl treated nets and 9.0% for the lambdacyhalothrin treated nets (P < 0.01). No such effect was observed against *Culex*, the proportion found inside the nets showing little variation (8–9%) between the untreated nets and nets treated with chlorpyrifos-methyl or lambdacyhalothrin.

The mortality was consistently high among *A. gambiae *and *Culex *regardless of the treatment used. An application rate of 100 mg/m^2 ^chlorpyrifos-methyl was sufficient to kill 58% of *A. gambiae *and 68% of *Culex *(despite the fact that many of the mosquitoes carried genes for *Ace.1*^*R*^), and reduced the rate of blood feeding through the holed nets by 62% among *A. gambiae *and 88% among *Culex*. An increase in the application rate of chlorpyrifos-methyl from 100 to 250 mg/m^2 ^did not significantly increase the mortality rates of *A. gambiae *and *Culex*, but with the higher dose there was complete inhibition of blood feeding of *A. gambiae*. Most of the mortality was evident by dawn and for most types of treatment less than 10% of deaths occurred over the next 24 h. The two-in-one and the high and low dose mixtures showed equivalent efficacy. When compared to each insecticide applied as single treatments the two-in-one and high dose mixture did not appear to give either better protection against blood feeding or higher mosquito mortality. The low dose mixture was associated with mortality and blood-feeding rates similar to that observed with the high dose mixture and two-in-one treatments.

All treatments increased the proportion of mosquitoes in the verandah traps. The magnitude of the excito-repellent effect ranged from 16% to 40%. By this criterion, lamdacyhalothrin and chlorpyrifos-methyl were equally repellent, and both *Culex *and *A. gambiae *showed a similar tendency to be repelled. With the greater numbers of mosquitoes collected, the "insecticide-induced exophily" tended to be significant for *Culex*, but it was not significant with the small *A. gambiae *samples.

### Side-effects of treatments

In 154 interviews with sleepers recorded during the first two weeks of the trial, 14 (9.1%) recorded sneezing and 5 five recorded severe headaches. Such symptoms occurred during the first 10 days after treating the nets. The complaints of headache were made, in every instance, after a night spent under the unwashed net treated with 250 mg/m^2 ^chlorpyrifos-methyl.

### Genotype frequencies

The frequency of the *kdr *gene in *A. gambiae *from the control huts was above 90%. With this high frequency the sample sizes were considered too low to test satisfactorily for any selective effect by treatments, and, therefore, the collections from the treatment huts were not tested any further for *kdr*. The high mortality obtained with the lambdacyhalothrin treated nets indicates that this pyrethroid, when present on nets, is still effective in controlling many *kdr *resistant mosquitoes.

The results of *Ace.1 *genotyping of 298 *A. gambiae *are grouped into those found live or dead in the huts with untreated control nets, or with nets treated with chlorpyrifos-methyl, lambdacyhalothrin or combinations of these two insecticides (Table 2). The numbers identified were two mosquitoes with *Ace.1*^*R *^only, 243 mosquitoes with *Ace.1*^*R *^plus *Ace.1*^*S *^and 53 mosquitoes with *Ace.1*^*S *^only. Assuming that the first type were homozygous for *Ace.1*^*R*^, the third type were homozygous for *Ace.1*^*S *^and the second type were heterozygous, the frequencies of the *Ace.1*^*R *^and *Ace.1*^*S *^alleles would be 0.41 and 0.59 respectively. According to conventional Hardy-Weinberg ratios (P^2^, 2PQ, Q^2^) the frequency of *Ace.1*^*R*^*/Ace.1*^*R *^genotype was expected to be 17.2% rather than the 0.7% actually observed. This indicates a strong fitness disadvantage associated with *Ace.1*^*R *^homozygotes or some alternative genetic explanation.

**Table 2:** Acetylcholinesterase genotypes as determined by PCR on *Anopheles gambiae *collected from the experimental huts.

Type of Treatment	Live/dead at collection	*Ace*1^R^/*Ace1*^R ^No.	*Ace*1^R^/*Ace*1^S^		*Ace*1^S^/*Ace*1^S^	
				
			No.	% survival	No.	% survival
Untreated Control	live	0	27	64%^a^	9	100%^b^
	dead	0	15		0	
	total	0	42		9	

						
Chlorpyrifos- methyl	live	1	23	26%^c^	11	46%^c^
	dead	1	65		13	
	total	2	88		24	

						
Lambda- cyhalothrin	live	0	16	34%^c^	2	14%^c^
	dead	0	31		12	
	total	0	47		14	

						
Mixture & two-in-one	live	0	21	47%^c^	1	17%^c^
	dead	0	45		5	
	total	0	66		6	

Note percentages accompanied by different letter superscripts differ significantly by χ^2 ^or Fisher's exact test

Survival rates of *Ace.1*^*R*^*/Ace.1*^*S *^in huts with untreated nets were lower than that of *Ace.1*^*S*^*/Ace.1*^*S *^suggesting a fitness disadvantage. There was no apparent difference between the survival rates of *Ace.1*^*R*^*/Ace.1*^*S *^and *Ace.1*^*S*^*/Ace.1*^*S *^in huts with chlorpyrifos-methyl treated nets or other treatments.

## Discussion

Where ITN-induced deterrency occurs, it will contribute to reducing human/vector contact, and in households where not everyone has access to a net, even non-users would be expected to gain some indirect protection from mosquito biting. However, deterrency will reduce the number of mosquitoes killed and, hence, the potential for a community-wide impact on the infective biting population. Thus, the mean numbers found fed and dead in the huts are more realistic indicators of the relative impact of the treated nets than are the columns showing the proportions fed and dead. However, the latter are also of interest in giving an idea of the extent of the feeding inhibition and insecticidal effects of the various treatments on mosquitoes exposed to them.

Nets treated with chlorpyrifos-methyl appeared to induce a level of deterrency against *A. gambiae *similar to that of the pyrethroid lambdacyhalothrin but the effects were not statistically significant. Compared to other hut studies in Côte d'Ivoire, the deterrency shown by chlorpyrifos-methyl against *C. quinquefasciatus *was greater than that shown by Olyset nets [12] or by nets treated with permethrin 500 mg/m^2 ^[32] or carbosulfan 200 and 300 mg/m^2 ^[13,14,25]. The deterrency of chlorpyrifos-methyl was still evidence presently after five washes. This contrasts with another type of OP, pirimiphos -methyl which was undetectable on nets after only 3 three washes [33]. The difference partly lies with the formulations used. The microencapsulated formulation was specially developed to bind chlorpyrifos-methyl more strongly and to release it more gradually compared to the emulsifiable concentrate used with pirimiphos -methyl. The manufacturer of chlorpyrifos-methyl (Dow AgroSciences) has recently improved the release characteristics and wash-fastness of the microencapsulation so that insecticide persistence and performance compares favourably with pyrethroids and DDT (Rowland & Yates, unpublished). The microencapsulated formulation used for lambdacyhalothrin has been shown earlier to withstand at least 5 washes [21,34] and this was re-confirmed in the present trials.

Only a small proportion of mosquitoes in the hut with the intact, untreated net had blood-fed. The protective effect of untreated nets was lost when they were holed. This re-confirms the results in experimental huts of Lines et al [35] and Curtis et al [20] and data on malaria in children sleeping under untreated nets which were either intact or torn [36]. It emphasizes the point that ITN programmes which fail to ensure that nets are and remain effectively insecticidal cannot expect to achieve an impact on malaria when the nets become torn, as they inevitably will.

Treatment with chlorpyrifos-methyl or lambdacyalothrin restored the protectiveness of torn nets against *Culex *and *A. gambiae*, as has been demonstrated with various pyrethroid treatments [20,32,35,37].

Whether or not *A. gambiae *feeds through chlorpyrifos-methyl treated nets seems to depend upon the concentration of insecticide used, the observed blood feeding rate being zero for the net treated with 250 mg/m^2 ^and 14.6% with 100 mg/m^2^. Further tests with intermediate concentrations would be needed to confirm a dosage trend. Mortality also appeared to be dosage-dependent, but the difference between the doses was non-significant among the small numbers collected.

Adverse effects involving headache and sneezing were associated with exposure to nets recently treated with 250 mg/m^2 ^chlorpyrifos-methyl. Such effects were not apparent with nets treated with 100 mg/m^2 ^chlorpyrifos-methyl or with washed nets or with nets used two weeks after treatment. Symptoms appeared to be dosage-dependent. With lambdacyhalothrin treated nets, sneezing was reported with a 30 mg/m^2 ^dosage [38] and 20 mg/m^2 ^[21] but not at 10 mg/m^2 ^[38] or with the dosage of lambdacyhalothrin used in the present trial (18 mg/m^2^). While the toxicological profile of chlorpyrifos-methyl is favourable, a more comprehensive hazard assessment is warranted before considering community trials [39,40]. To avoid the side effects which were reported at 250 mg/m^2^, it is proposed that a dose of 100 mg/m^2 ^should not be exceeded.

Resistance due to insensitive acetylcholinesterase has arisen independently through a point mutation to G119S (glycine to serine substitution) of the *Ace.1 *gene on several occasions in *A. gambiae*, *Anopheles albimanus*, *C. quinquefaciatus *and *Culex pipiens *[31]. This study confirms the finding of Weill et al [31] on the same population of *A. gambiae *of there being far fewer homozygotes for the *Ace.1*^*R *^than would be expected from the Hardy Weinberg ratio, indicating extremely low viability of these homozygotes. In the huts with untreated nets, the observed survival of heterozygotes for this gene was significantly less than that of homozygotes for *Ace.1*^*S*^. With such strong selection pressure against *Ace.1*^*R *^at the adult stage in homozygotes and heterozygotes, this gene could apparently only persist in the population if there is very strong selection against *Ace.1*^*S *^in the immature stages. A more likely explanation is one of gene duplication having occurred at the acetylcholinesterase locus. Such duplication of the *Ace.1 *locus is common in *C. pipiens *[31], and the existence of such duplication in *A. gambiae *could result in *Ace.1*^*S *^and *Ace.1*^*R *^being present at diffent loci on the same chromosome. Insects with one locus for *Ace.1*^*S *^and one for *Ace.1*^*R *^could be included among those labelled *Ace.1*^*R*^/*Ace.1*^*S *^in table 4. The rare individuals labelled *Ace.1*^*R*^/*Ace.1*^*R *^in Table 2 would be those where both *Ace.1 *loci and all alleles were of the R type.

The survival of all genotypes was significantly lower in huts with treated nets than with untreated nets, and the survival of mosquitoes with at least one *Ace.1*^*R *^allele and those with only *Ace.1*^*S *^did not differ significantly on any of the treatments. Thus under the realistic conditions of the experimental huts the *Ace.1*^*R *^allele does not give effective resistance to chlorpyrifos-methyl.

The present study is the first experimental hut trial of chlorpyrifos-methyl on nets. Other recent trials with non-pyrethroids have focused on carbosulfan which, like chlorpyrifos-methyl, is an acetylcholinesterase inhibitor known to be effective against *kdr *resistant *A. gambiae *and *C. quinquefasciatus *[13-15,25]. Quite apart from its potentially toxic breakdown product, carbofuran [25], carbosulfan has the disadvantage of poorer wash fastness compared to alphacyano-pyrethroids [14]. The rate of conversion of carbosulfan to carbofuran and the hazard this presents to net users has yet to be established, and the inferior wash fastness might conceivably be improved through formulation technology. But in view of these uncertainties, chlorpyrifos-methyl seems the better prospect at the present time. Chlorpyrifos-methyl should be subjected to a full hazard assessment before being used on a community scale [40].

There are several potential advantages to using combinations of insecticide on nets: a) a reduced or delayed selection of resistance alleles, b) an improved control of resistant populations, c) an improved efficacy if the two components are synergistic, with possible cost savings and improved safety if the dosage can be substantially reduced as a result. To delay the selection of resistance with a mixture would require no cross-resistance between the two components and 100% mortality of insects carrying resistance to one of the components on being exposed simultaneously to the other component [23,41]. The situation in the Bouaké region of Côte d'Ivoire is not favourable to classical resistance management of genes that have an impact on chemical control, because *kdr *and *Ace.1*^*R *^are already present at high frequency. Elsewhere in Africa, where neither mechanism is present a strategy of using mixtures of lambdacyhalothrin and chlorpyrifos-methyl might have potential for resistance management. There is some evidence in laboratory studies for synergy between pyrethroids and OPs/carbamates against susceptible *A. gambiae *adults [42] and resistant *C. quinquefasciatus *larvae bearing *kdr *and elevated oxidase mechanisms [43], but not against adults with site-insensitivity resistance mechanisms [44]. The encouragingly high efficacy of the low dosage mixture could be due to synergism, but to test this hypothesis a comparison should be made in experimental huts with the low dosages applied individually to nets.

Both chlorpyrifos-methyl and lambdacyhalothrin treated nets and combinations of these insecticides were shown in the realistic conditions of experimental huts to be equally effective in preventing blood feeding by, and killing of, *A. gambiae *and *C. quinquefasciatus*, which laboratory and molecular studies suggested would have shown pyrethroid and organophosphate resistance. Thus, these resistance genes may have little or no practical significance. To demonstrate this unequivocally, it is desirable to examine the impact of mixtures and single insecticide treatments on both malaria transmission and the selection of resistance alleles in an area where resistance to OPs and pyrethroids in *A. gambiae *and *C. quinquefasciatus *exist at low-to-intermediate frequencies. Chlorpyrifos-methyl should also be tested on nets and as an indoor residual spray treatment against populations of pyrethroid resistant *C. quinquefasciatus *(in Tanzania) and *A. funestus *(in South Africa), respectively, that have demonstrated practical impact in the field [6,21]. Chlorpyrifos-methyl could be useful for managing such problematic examples of resistance if it could be shown that these pyrethroid resistance mechanisms confer no cross- resistance to organophosphates.

## Authors' contributions

ANA & RNG conducted the field work, summarizsed the data, and wrote the first report.

AAK, FC, VC conducted or supervised the genotyping and interpreted the results.

MWR designed the study, supervised RNG and ANA, analysed the data and wrote the manuscript.

CFC co-designed the study, co-supervised ANA and contributed to the manuscript.

JMH co-designed the study and supervised FC and VC and contributed to the manuscript.

FD designed the experimental huts.

MZ established the collaboration with Dow AgroSciences, coordinated the evaluation of chlorpyrifos-methyl, and contributed to study design and writing of the manuscript.

## Supplementary Material

Additional File 1 Summary data of *Anopheles gambiae *collected from experimental huts over 33 nights at Yaokoffikro. Click here for file

Additional File 2 Summary data of *Culex *spp. collected from experimental huts over 33 nights at Yaokoffikro. Click here for file
