# Supplementary material for: Experimental hut evaluation of bednets treated with an organophosphate (chlorpyrifos-methyl) or a pyrethroid (lambdacyhalothrin) alone and in combination against insecticide-resistant Anopheles gambiae and Culex quinquefasciatus mosquitoes
Source: Malar J. 2005 May 26;4:25. doi: 10.1186/1475-2875-4-25 (PMC1156935; doi:10.1186/1475-2875-4-25)
Supplement: Additional File 1 — Summary data of Anopheles gambiae collected from experimental huts over 33 nights at Yaokoffikro. [file 1475-2875-4-25-S1.doc]

**Additional file 1. Summary data of *Anopheles gambiae* collected from experimental huts over 33 nights at Yaokoffikro. Numbers in the same column sharing a letter superscript do not differ significantly (P>0.05).**

| Treatment | Dose (mg/m^2^) | Total number | % deterred | % inside net | % bloodfed | 95% C.I. | % feeding inhibition | Mean number fed per night (rank) | % dead after 24h | 95% C.I. | Mean number dead per night  (rank) | % in exit trap | 95% C.I. |
| --- | --- | --- | --- | --- | --- | --- | --- | --- | --- | --- | --- | --- | --- |
| Control * | Untreated | 123^a^ |  | 3.3 | 7.3^af^ | (3.9-13.5) |  | 0.27^ab^ (10) | 17.1^a^ | (11.4-24.8) | 0.64^ab^ (3) | 35.0^a^ | (27.1-43.8) |
| Control | Untreated | 83^b^ |  | 20.5 | 38.6^be^ | (28.4-49.4) |  | 0.97^a^ (11) | 10.8^a^ | (5.7-19.5) | 0.27^a^ (1) | 42.2^ab^ | (32.0-53.0) |
| Chlorpyrifos-methyl | 250 | 36^bc^ | 56.6 | 5.6 | 0^d^ | (0-9.2) | 100 | 0.00^c^ (1) | 69.4^bc^ | (52.8-82.2) | 0.76^b^ (6) | 47.2^abc^ | (31.7-63.3) |
| Chlorpyrifos- methyl | 250** | 37^bc^ | 55.4 | 18.9 | 21.6^bef^ | (11.2-37.6) | 44.0 | 0.24^ab^ (9) | 73.0^bc^ | (56.7-84.8) | 0.82^b^ (7) | 40.5^ab^ | (26.1-56.8) |
| Chlorpyrifos- methyl * | 100 | 48^bc^ | 42.2 | 10.4 | 14.6^af^ | (7.1-27.6) | 62.2 | 0.21^ac^ (8) | 58.3^c^ | (44.1-71.3) | 0.85^b^ (8) | 52.1^bc^ | (38.2-65.7) |
| Chlorpyrifos-methyl | 100 | 50^bc^ | 59.3 | 0 | 6.0^a^ | (1.9-17.0) | 84.4 | 0.09^ac^ (4) | 62.0^bc^ | (48.0-74.3) | 0.94^b^ (10) | 56.0^bc^ | (42.1-69.0) |
| Lambdacy-halothrin | 18 | 34^bc^ | 59.0 | 8.8 | 0^cd^ | (0-10.3) | 100 | 0.00^c^ (1) | 67.6^bc^ | (50.5-81.1) | 0.70^b^ (4) | 58.8^bc^ | (41.9-73.9) |
| Lambdacy-halothrin | 18** | 24^c^ | 71.1 | 8.3 | 16.7^ae^ | (6.4-36.9) | 56.7 | 0.12^abc^ (5) | 75.0^bc^ | (54.4-88.3) | 0.55^ab^ (2) | 58.3^bc^ | (38.3-75.9) |
| Two-in-one | 100 CM, 18 L | 34^c^ | 59.0 | 8.8 | 2.9^a^ | (0.4-18.1) | 92.5 | 0.03^c^ (3) | 67.6^bc^ | (50.5-81.1) | 0.70^ab^ (4) | 55.9^bc^ | (39.2-71.4) |
| Mixture | 100 CM, 18 L | 38^bc^ | 54.2 | 5.3 | 13.2^af^ | (5.6-28.1) | 65.8 | 0.15^abc^ (6) | 78.9^b^ | (63.2-89.1) | 0.91^b^ (9) | 44.7^abc^ | (29.9-60.5) |
| Mixture | 25 CM, 4.5 L | 58^b^ | 30.1 | 10.3 | 10.3^af^ | (4.7-21.2) | 73.3 | 0.18^abc^ (7) | 67.2^bc^ | (54.3-78.0) | 1.18^b^ (11) | 62.1^c^ | (49.1-73.6) |

* intact net (unholed); all others had 80 holes cut in them to simulate damaged nets

** washed 5 times
