# Supplementary material for: Experimental hut evaluation of bednets treated with an organophosphate (chlorpyrifos-methyl) or a pyrethroid (lambdacyhalothrin) alone and in combination against insecticide-resistant Anopheles gambiae and Culex quinquefasciatus mosquitoes
Source: Malar J. 2005 May 26;4:25. doi: 10.1186/1475-2875-4-25 (PMC1156935; doi:10.1186/1475-2875-4-25)
Supplement: Additional File 2 — Summary data of Culex spp. collected from experimental huts over 33 nights at Yaokoffikro. [file 1475-2875-4-25-S2.doc]

**Additional file 2. Summary data of *Culex* spp. collected from experimental huts over 33 nights at Yaokoffikro. Numbers in the same column sharing a letter superscript do not differ significantly (*P*>0.05).**

| Treatment | Dose (mg/m^2^) | Total number | % deterred | % inside net | % bloodfed | 95% C.I. | % feeding inhibition | Mean number fed per night (rank) | % dead after 24h | 95% C.I. | Mean number dead per night (rank) | % in exit trap | 95% C.I. |
| --- | --- | --- | --- | --- | --- | --- | --- | --- | --- | --- | --- | --- | --- |
| Control* | Untreated | 435^a^ |  | 0.2^q^ | 2.1^ac^ | (1.1-3.9) |  | 0.27^a^ (9) | 9.4^a^ | (7.0-12.6) | 1.24^a^ (2) | 41.6^a^ | (37.1-46.3) |
| Control | Untreated | 352^a^ |  | 8.2^b^ | 25.0^b^ | (20.8-29.8) |  | 2.67^b^ (11) | 9.7^a^ | (7.0-13.2) | 1.03^a^ (1) | 38.9^a^ | (34.0-44.1) |
| Chlorpyrifosmethyl | 250 | 196^bc^ | 44.3 | 10.7^b^ | 2.0^ac^ | (0.8-5.3) | 92.0 | 0.12^ac^ (3) | 76.0^bd^ | (69.5-81.5) | 4.52^b^ (9) | 45.9^ab^ | (39.1-52.9) |
| Chlorpyrifos methyl | 250** | 225^b^ | 36.1 | 10.2^b^ | 4.0^a^ | (2.1-7.5) | 84.0 | 0.27^a^ (9) | 65.3^c^ | (58.2-71.3) | 4.45^b^ (8) | 50.7^b^ | (44.2-57.2) |
| Chlorpyrifosmethyl* | 100 | 200^bc^ | 43.2 | 8.0^b^ | 3.0a\ | (1.4-6.5) | 88.0 | 0.18^ac^ (7) | 68.5^bc^ | (61.7-74.6) | 4.15^b^ (7) | 50.0^b^ | (43.1-56.9) |
| Chlorpyrifosmethyl | 100 | 203^bc^ | 53.3 | 0^a^ | 2.0^ac^ | (0.7-5.1) | 92.0 | 0.12^ac^ (3) | 65.0^c^ | (58.2-71.3) | 4.00^b^ (6) | 53.7^b^ | (46.8-60.4) |
| Lambdacy-halothrin | 18 | 148^c^ | 57.9 | 9.5^b^ | 4.1^a^ | (1.8-8.7) | 83.6 | 0.18^ac^ (7) | 66.9^cd^ | (58.9-74.0) | 3.00^b^ (3) | 54.1^b^ | (46.0-61.9) |
| Lambdacy-halothrin | 18** | 213^bc^ | 39.5 | 12.7^b^ | 0.5^c^ | (0.1-3.3) | 98.0 | 0.12^ac^ (3) | 70.0^bc^ | (63.5-75.7) | 4.52^b^ (9) | 46.0^ab^ | (39.4-52.7) |
| Two-in-one | 100 CM, 18 L | 193^bc^ | 45.2 | 8.3^b^ | 1.6^ac^ | (0.5-4.7) | 93.6 | 0.09^c^ (1) | 62.7^c^ | (55.7-69.2) | 3.67^b^ (5) | 54.4^b^ | (47.3-61.3) |
| Mixture | 100 CM, 18 L | 211^bc^ | 40.0 | 8.1^b^ | 1.4^ac^ | (0.5-4.3) | 94.4 | 0.09^ac^ (1) | 74.9^bd^ | (68.6-80.3) | 4.79^b^ (11) | 46.9^ab^ | (40.3-53.7) |
| Mixture | 25 CM, 4.5 L | 186^bc^ | 47.1 | 10.8^b^ | 2.7^ac^ | (1.1-6.3) | 89.2 | 0.15^ac^ (6) | 61.8^c^ | (54.6-68.5) | 3.48^b^ (4) | 45.2^ab^ | (38.2-52.4) |

* intact net (unholed); all others had 80 holes cut in them to simulate damaged nets

** washed 5 times
